# Salivary cortisol, dehydroepiandrosterone, and chromogranin A levels in patients with gingivitis and periodontitis and a novel biomarker for psychological stress

**DOI:** 10.3389/fendo.2023.1147739

**Published:** 2023-04-11

**Authors:** Yeon-Hee Lee, Chon Suk, Seung-Il Shin, Ji-Youn Hong

**Affiliations:** ^1^ Department of Orofacial Pain and Oral Medicine, Kyung Hee University Dental Hospital, Kyung Hee University, Seoul, Republic of Korea; ^2^ Department of Endocrinology, Kyung Hee University Medical Center, Kyung Hee University, Seoul, Republic of Korea; ^3^ Department of Periodontology, Periodontal-Implant Clinical Research Institute, School of Dentistry, Kyung Hee University, Seoul, Republic of Korea

**Keywords:** saliva, periodontitis, cortisol, chromogranin A, stress, biomarkers

## Abstract

**Introduction:**

This study aimed to investigate the neuroendocrine responses based on cortisol, dehydroepiandrosterone (DHEA), cortisol/DHEA ratio, and chromogranin A levels, which reflect the activity of the hypothalamic-pituitary-adrenal axis, according to the presence or absence of psychological stress in patients with gingivitis and periodontitis compared to that in healthy controls.

**Methods:**

In total, 117 patients (60 women, mean age: 36.29 ± 19.03 years) participated in this case-control study, comprising 32 healthy controls, 49 patients with gingivitis, and 36 patients with periodontitis. We investigated the presence of psychological stress and salivary characteristics, and analyzed the stress-related biomarkers of cortisol, DHEA, cortisol/DHEA ratio, and chromogranin A in the stimulated saliva.

**Results:**

Salivary cortisol and chromogranin A levels increased with the severity of periodontal disease; their levels were the highest in the periodontitis group and were significantly higher in the following descending order: periodontitis, gingivitis, and healthy control groups (all values of p < 0.001). Additionally, the DHEA levels and cortisol/DHEA ratio were higher in the periodontitis group than those in the healthy control group (all values of p < 0.001). A multivariate logistic regression analysis revealed that the factors predicting above-average cortisol levels were periodontitis (odds ratio [OR] = 256.829; p < 0.001), women (OR = 6.365; p = 0.004), and psychological stress (OR = 6.036; p = 0.007); those predicting above-average cortisol/DHEA ratios were periodontitis (OR = 11.436; p < 0.001), psychological stress (OR = 3.977; p = 0.003), and women (OR = 2.890; p = 0.026). Thus, periodontitis and psychological stress were significant and strong predictors of above-average cortisol levels and cortisol/DHEA ratios. In the gingivitis group, salivary cortisol levels (r = 0.381, p = 0.007) and cortisol/DHEA ratios (r = 0.479, p < 0.001) were correlated with the presence of psychological stress. In the periodontitis group, increased cortisol/DHEA ratios (r = 0.412, p = 0.013) and lowered salivary buffer capacities (r = -0.334, p = 0.047) were correlated with the presence of psychological stress.

**Conclusion:**

Periodontitis is a multifactorial disease resulting in inflammatory tissue destruction, which differs from gingivitis and a healthy state. Differences in stress-related neuroendocrine markers were revealed based on the severity of periodontal disease. The biomarkers that could be classified according to disease severity were salivary cortisol and chromogranin A levels. Above-average cortisol levels and cortisol/DHEA ratios are significant predictors of psychological stress in patients with gingivitis and periodontitis.

## 1 Introduction

Periodontal diseases are the most common oral diseases and are characterized by an inflammatory disease of the supportive apparatus surrounding the tissues of the teeth, which causes progressive degeneration the periodontium, including the gingival tissue, periodontal ligament, alveolar bone, and cementum (1). Periodontal diseases are mainly divided into gingivitis and periodontitis, and the critical characteristic to discriminate between the two is the loss of the supporting connective tissue and alveolar bone. Gingivitis is the mildest form, occurring in up to 90% of the population (2). Additionally, gingivitis is a reactive disease that can be reversed by improving oral hygiene but if left untreated, it can progress to its more severe form, periodontitis; the latter is characterized by chronic inflammation and can cause irreversible damage to the periodontium and the loss of bone or periodontal support (3).

Both gingivitis and periodontitis are associated with dysbiosis of oral microbiome in the biofilm as the microbiota imbalance plays critical roles in host-damaging immune-inflammatory response along with the disease development (4). Accumulation of dental plaque brings out a shift in the composition of dominant species in the subgingival biofilm from health to gingivitis with an increase in bacterial biomass. Microbial species commonly associated with gingivitis are enriched with gram-negatives such as *Prevotella* spp., *Treponema* sp., *Fusobacterium* spp., and *Selenomonas* spp. (5, 6). Periodontitis is accompanied by profound shifts to diverse groups of gram-negative species including red-complex triad comprised of *Porphyromonas gingivalis*, *Tannerella forsythia and Treponema denticola* (7). Keystone pathogens such as *P. gingivalis* interfere with complement-mediated host immune response, trigger imbalance between host and bacterial interaction and further establish dysbiosis (8). Microenvironmental changes by keystone pathogens might enhance growth of additional pathobionts which stimulate immune response and severe the disease progression. However, the critical pathogenic mechanism responsible for the transition of gingivitis to periodontitis is still unclear, and the knowledge of the differences between the two diseases is extensive. Based on a recent National Health and Nutrition Examination Survey conducted from 2009 to 2012 in the United States of America, the prevalence of periodontitis in adults ≥30-year-old was 46% (9). According to the national data from Korea, the prevalence of periodontitis in individuals ≥19 years of age was 23.9 and 32.9% in 2008 and 2010, respectively, increasing to 52% in those aged 65–74 years (Furuta et al.).

Periodontitis is accompanied by tissue destruction resulting from complex host immune-inflammatory reaction in response to the plaque bacteria (10). Multiple risk factors are known to be involved in the progression of periodontitis such as genetics, tobacco, viral infection, nutrition, diabetes, systemic diseases and stress (11–14), which might affect the susceptibility of host. Psychological stress has been consistently suggested as one of these factors (12, 15, 16). A direct relationship between periodontal disease and psychological stress has not been established owing to the lack of adequate clinical trials, the difficulty in quantifying the amount and duration of psychological stress, and several factors affecting the incidence and severity of periodontal disease (17). However, emotional stress disrupts homeostasis maintained by a complex network linking the endocrine, nervous, and immune systems, which can alter immune function and exacerbate periodontitis (18).

However, current studies suggest a positive relationship between stress and periodontitis with putative roles of stress as a risk factor for periodontitis (19). Neurobiological links between stress and periodontitis include activation of adrenergic nerve signaling axis that affects vasculature to decrease blood flow, reduce wound healing and immune response (20), and activation of hypothalamic-pituitary-adrenal (HPA) axis that subsequently increases the release of adrenocorticotropin and glucocorticoids particularly cortisol (21). Chronic dysregulation of cortisol level disrupts the body homeostasis with notable increase in the proinflammatory cytokine and immune system dysfunction which might worsen periodontitis. Chronic stress might induce physically adaptive changes in hippocampal morphology through epigenetic modifications, which decline health-related behaviors and lead to the health-damaging behaviors such as smoking and alcohol consumption (22). Prolonged higher level of cortisol from chronic stress might also be related to dysbiosis in oral microbiome and reduction of immune cell activity (23).

The relationship between periodontitis and psycho-neuro-immunological parameters, such as psychological stress, cortisol, dehydroepiandrosterone (DHEA), and chromogranin A, has rarely been studied (24). Cortisol, the major glucocorticoid in humans, is released when the HPA axis is activated by psychological stress, thus increasing glucose levels through gluconeogenesis and blood pressure (25). Furthermore, cortisol levels are the most representative stress biomarker, wherein salivary cortisol and blood cortisol levels are frequently used as biomarkers of psychological stress (26). A major reason for the extensive application of saliva sampling lies in its convenience as it is an easy-to-handle, non-invasive method. Long-term stress can lead to the dysregulation of the HPA axis that induces abnormal basal and stress levels of HPA hormones, including cortisol and DHEA (27). DHEA is a biomarker that requires approximately 1 h to increase in response to acute stress, and it increases after acute psychological stress irrespective of the type and duration of psychological stress (28). In particular, researchers have shown that increases in DHEA levels were greater in women, adolescents, and obese individuals, and blood and saliva measurements of DHEA were significantly correlated (29). Chromogranin A has recently received considerable attention for its role in stress-related diseases, although studies reporting a correlation between psychological health and salivary chromogranin A levels are scarce (30). Therefore, the direct measurement of psychological stress through salivary cortisol, DHEA, and chromogranin A levels is required in patients with periodontal diseases, such as gingivitis and periodontitis.

The oral microbiome is a key factor in the development and progression of periodontitis. The oral cavity likely harbors at least 600 bacterial species (31), and the bacterial plaques attached to teeth surfaces can also contain billions of oral bacteria (32). The influencing factors for various forms of periodontal diseases include local (i.e., because of plaque accumulation and oral hygiene habits), systemic, behavioral, and environmental factors (e.g., emotional stress and smoking habits) (33). The effects of psychological stress on the immune system and inflammatory responses are well known (34), and such stress may contribute to chronic inflammatory diseases, such as periodontitis. However, few scholars have demonstrated the relationship between the latter inflammatory disease and psychological stress (35), and a limited number of studies have evaluated the associations between or the influence of neuroendocrine responses and psychological factors on the severity and level of periodontal diseases. Therefore, existing literature has not yet evidenced on the direct link among periodontal disease, psychological stress, stress-related changes in the endocrine system, and HPA axis. Investigating this matter is challenging because of the multiple variables affecting the severity and level of periodontal diseases and the uncertainties surrounding qualitative and quantitative measures of stress in patients with periodontal diseases. In this study, we hypothesize that psychological stress induces the hyperactivation of the HPA axis and contributes to the onset and exacerbation of periodontitis.

In this study, the null hypothesis was that the activity of the HPA axis related to psychological stress in patients with gingivitis, periodontitis and healthy control will show no differences among the groups. Therefore, we directly measured salivary cortisol, DHEA, and chromogranin A levels and cortisol/DHEA ratio in patients with periodontal diseases (i.e., gingivitis and periodontitis) and derived the relationship among these stress-related neuroendocrine parameters according to the presence or absence of psychological stress. Ultimately, this study aimed to investigate the most powerful predictive biomarkers of psychological stress in patients with gingivitis and periodontitis compared with those in healthy controls.

## 2 Materials and methods

### 2.1 Study population

For this case-control study, patients were recruited through in-hospital advertisements at Kyung Hee University Dental Hospital (Seoul, Korea) from May 1, 2021 to October 31, 2022. The inclusion criteria were as follows: Korean adults aged >20 years; ≥20 remaining natural teeth, excluding wisdom teeth, at the time of the first examination; and healthy periodontium, gingivitis, or chronic periodontitis diagnoses (all defined below). Participants were classified based on full mouth records of clinical parameters, including the probing depth (PD), clinical attachment loss (CAL), and bleeding on probing (BOP), which were measured at six sites per tooth with a periodontal probe (Probe UNC 15; Hu-Friedy). The periodontal assessments were conducted by two experienced periodontists (JYH and SIS), and radiographic examinations were conducted to confirm the diagnosis for each participant. Calibration exercises by measuring CAL in 10 patients with 24-hour interval were conducted by two trained examiners. Intra-class correlation coefficients (ICCs) of 0.80 and 0.89 were estimated in intra-examiner reproducibility measurements. Inter-examiner ICCs for CAL were 0.80 and 0.81 in each measurement. When there was a disagreement, a unified conclusion was made through several discussions until a consensus was reached.

The case definition of healthy periodontium, gingivitis, and chronic periodontitis was based on criteria defined in the 2017 World Workshop on the Classification of Periodontal Diseases (36). A clinically healthy periodontium was diagnosed when the subject showed a PD ≤ 3 mm, BOP sites < 10%, and no CAL. A healthy control was defined as an individual who did not have any major systemic disease or was not regularly ingesting medication for physical/psychological problems. Participants with BOP sites ≥ 10% and PD ≤ 3 mm in all sites were diagnosed as gingivitis and included in the respective group. The periodontitis group comprised participants with: 1) interdental CAL of > 5 mm at the site of greatest loss; 2) radiographic bone loss of more than mid 1/3 of the root; 3) tooth loss because of periodontal disease; and 4) a maximum PD of ≥ 6 mm that affected ≥ 30% of teeth, which correspond to stages III and IV in the generalized pattern. The exclusion criteria were described as follows: history of periodontal treatment within the last 6 months; having taken medications that may affect periodontal conditions (antibiotics, anti-inflammatory drugs, anticonvulsants, immunosuppressants, calcium channel blockers, etc.) within the last 6 months; taking anticoagulants (e.g., aspirin); have uncontrolled systemic diseases; pregnant or breastfeeding; and 6) the presence of intraoral appliances as part of orthodontic treatment. For the sample size calculation, we used the G*Power software (latest ver. 3.1.9.7; Heinrich-Heine-Universität Düsseldorf, Düsseldorf, Germany); 89 subjects were included with an actual target of at least 30 per group as suitable for statistical analysis, and a total of 117 participants were recruited. All participants were given information about the study and provided informed consent, and all protocols were approved by the Committee on Ethics of the Kyung Hee Clinical Research Institute, Kyung Hee University Medical Center (IRB no. KH-DT20030).

### 2.2 Study design

All patients underwent panoramic radiography for the diagnosis of periodontitis. Through a well-formed questionnaire, the presence or absence of smoking habits and daily psychological stress were identified using a dichotomous question. As for the existence of psychological stress, participants answered yes/no to the question “Do you currently have daily psychological stress?” at the time of saliva collection. Unstimulated salivary flow rate (UFR), stimulated salivary flow rate (SFR), salivary pH, and salivary buffer capacity were measured to investigate salivary characteristics. Using saliva samples obtained through SFR measurement, we measured the levels of stress-related neuroendocrine markers, namely, cortisol, DHEA, cortisol/DHEA ratio, and chromogranin A. The detailed process is described below.

### 2.3 Saliva collection and salivary flow rate

Prior to the laboratory collection of saliva, participants were instructed to refrain from caffeine and/or nicotine for at least 4 h and alcohol for at least 24 h. Saliva was collected between 9:00 and 11:00. to minimize circadian differences. The UFR was obtained by measuring the amount of saliva collected using the spitting method for 10 min while the patient was resting. After a 2-min pre-stimulation period to remove saliva retained in the ducts, the SFR was determined by measuring the amount of saliva collected while chewing gum for 5 min with habitual chewing of 1 g of gum base. The UFR and SFR were expressed in mL/min (37).

### 2.4 Salivary pH and salivary buffer capacity

We used GC Saliva Check Buffer kits (GC Company) to determine the salivary pH and buffer capacity. The kits contained *in-vitro* pH test strips, saliva dispensing cups, wax gum pieces for saliva stimulation, saliva dispensing pipettes, buffer test strips, and a testing chart for determining the pH and saliva buffer capacity. After measuring UFR, a salivary pH test strip was inserted into the unstimulated whole saliva, which in turn was placed into the sample of resting saliva for 10 s. A pH value of 6.8 or higher corresponds to healthy saliva, whereas a value lower than 6.6 is characterized as acidic.

The saliva buffering capacity was measured using a pipette to drop the collected unstimulated whole saliva onto three areas of the test strip. After 2 min, the colors that appeared in the three areas were scored as follows: green, 4 points; green/blue, 3 points; blue, 2 points; red/blue, 1 point; and red, 0 points. The result was interpreted using the scheme in the kit, wherein each total value corresponds to a degree of salivary buffer capacity, as follows: 0–5, very low; 6–9, low; and 10–12, normal.

### 2.5 Assay of cortisol, DHEA, and chromogranin A

Salivary cortisol, DHEA, and chromogranin A were obtained from stimulated whole saliva samples. Human salivary cortisol was measured using radioenzyme immunoassay (Diagnostics Biochem Canada Inc.) using a commercial enzyme-linked immunosorbent assay (ELISA) kit according to the manufacturer’s instructions. We used an ELISA kit (MyBiosource) to measure the DHEA hormone and the Human Chromogranin A ELISA Kit (MyBiosource) to measure the chromogranin A levels. The saliva samples were kept on ice and stored at -20°C until analysis. On the day of analysis, saliva samples were centrifuged for 10 min at 2000 × g to remove particulate material. All samples were processed in triplicate during the same assay. Statistical analyses were conducted using the average of the three repeated measurements.

### 2.6 Statistics

The data were analyzed using the Statistical Package for the Social Sciences (SPSS) for Windows (version 26.0; IBM Corp.). Descriptive statistics were reported as mean ± standard deviation or numbers with percentages where appropriate. To analyze the distribution of discontinuous data, we used the χ2 and Bonferroni tests for the equality of proportions. Analysis of variance and Tukey’s *post-hoc* test were used to compare the values of parameters among the healthy control, gingivitis, and periodontitis groups. Spearman’s correlation analysis was used to determine factors associated with psychological stress and the relationships among stress-related neuroendocrine factors. The correlation coefficients (r) indicate the strength of the correlation and range between -1 and 1; the closer the absolute value of r is to 1, the stronger is the relationship.

Multivariate logistic regression analysis was conducted to assess whether any independent variable led to a significant increase in the probability of the two outcome variables (above-average cortisol level, cortisol/DHEA ratio, and chromogranin A level). Odds ratios (ORs) were calculated to determine how a unit change in a dependent variable affected the likelihood of the presence of an outcome variable, wherein ORs greater than 1 implied a greater relative risk. For all analyses, the statistical significance was set at a two-tailed p-value of < 0.05.

### 2.7 Ethics information

All participants were given information about the study and provided informed consent, and all protocols were approved by the Committee on Ethics of the Kyung Hee Clinical Research Institute, Kyung Hee University Medical Center (IRB no. KH-DT20030).

## 3 Results

### 3.1 Demographics and saliva characteristics

In total, 117 patients (60 women; mean age: 36.29 ± 19.03 years) participated in this study; their distribution is as follows: 32 healthy controls (18 women, 56.3%; 41.97 ± 13.48 years), 49 patients with gingivitis (22 women, 44.9%; 52.39 ± 14.98 years), and 36 patients with periodontitis (20 women, 55.6%; 52.39 ± 14.98 years). There was no significant difference in the distribution of sex by group, but the age individuals in the gingivitis and periodontitis groups was significantly higher than that of the healthy control group (p < 0.001). The prevalence of psychological stress was observed in 44.4% of patients with periodontitis, which was higher and lower than those in patients with gingivitis (40.8%) and healthy controls (59.4%), respectively, but these values were not significantly different (p = 0.245). The recent prevalence of psychological stress in Koreans has been reported 27.9–82.0% (38, 39). The distribution of psychological stress by sex indicated psychological stress in 33 women (55.0%) and 22 men (38.6%), respectively, and there was no significant sex-difference (p = 0.096).

Although 11.1% of the patients in the periodontitis group smoked, there was no significant difference between the smoking habits in the healthy control (3.1%) and gingivitis (6.1%) groups. The UFR (1.18 ± 0.37 mL/min) and SFR (1.55 ± 0.67 mL/min) of the healthy control group were not significantly different from those (UFR and SFR: 1.33 ± 0.50 and 1.56 ± 0.57 mL/min, respectively) of the periodontitis group. The average UFR and SFR values across groups showed that all participants had normal salivary flow rates.

Among the healthy control group, the salivary pH and salivary buffer capacity were 7.15 ± 0.41 and 10.69 ± 0.82, respectively, and the periodontitis group showed similar values (salivary pH: 6.96 ± 0.55; salivary buffer capacity: 10.25 ± 1.68). No significant differences were observed in salivary pH and salivary buffer capacity between groups, and their average values were within the normal range (**Table 1**).

**Table 1:** The demographic, clinical, and salivary characteristics of participants by group.

	Healthy control (n=32)	Gingivitis (n=49)	Periodontitis (n=36)	p-value	*post-hoc* analysis
	mean ± SD or n (%)	mean ± SD or n (%)	mean ± SD or n (%)		
Demographics					
**Age (years)** ^a^	41.97 ± 13.48	52.39 ± 14.98	52.39 ± 14.98	<0.001***	Healthy control <Gingivitis, Healthy control <Periodontitis
***Sex*** ^b^					
**Male**	14 (43.8%)	27 (55.1%)	16 (44.4%)	0.502	
**Female**	18 (56.3%)	22(44.9%)	20 (55.6%)		
Clinical characteristics					
**Psychological stress** ^b^	19 (59.4%)	20 (40.8%)	16 (44.4%)	0.245	
**Smoking habit** ^b^	1 (3.1%)	3 (6.1%)	4 (11.1%)	0.414	
Salivary characteristics					
**UFR (mL/min)** ^a^	1.18 ± 0.37	1.23 ± 0.26	1.33 ± 0.50	0.276	
**SFR (mL/min)** ^a^	1.55 ± 0.67	1.56 ± 0.51	1.56 ± 0.57	0.998	
**Salivary pH** ^a^	7.15 ± 0.41	7.02 ± 0.49	6.96 ± 0.55	0.274	
**Salivary buffer capacity** ^a^	10.69 ± 0.82	10.57 ± 0.74	10.25 ± 1.68	0.246	

a Analysis of variance and Tukey’s paired comparison test were used to compare the mean values among the three groups.

b Results were obtained using the two-sided chi-square test (two-sided). Statistical significance was set at a p < 0.05.

***: p < 0.001. SD, standard deviation; SFR, stimulated salivary flow rate; and UFR, unstimulated salivary flow rate.

### 3.2 Cortisol, DHEA, cortisol/DHEA ratio, and chromogranin A

**Figure 1:**
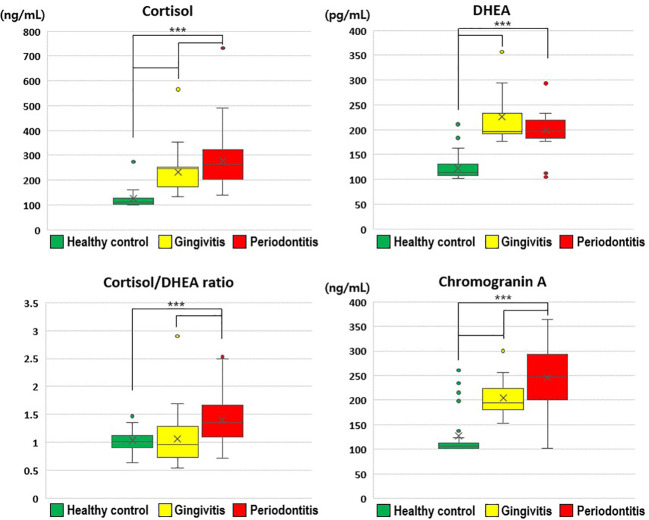
Comparisons of cortisol, dehydroepiandrosterone, cortisol/dehydroepiandrosterone ratio, and chromogranin A values by group. The results were obtained from ANOVA and Tukey’s paired comparison test. Statistical significance was set at p < 0.05. ***: p < 0.001. DHEA, dehydroepiandrosterone.

Salivary cortisol and chromogranin A levels increased with the severity of periodontal disease (**Figure 1**). In particular, the salivary cortisol level was significantly higher in the gingivitis group (231.52 ± 75.49 ng/mL) than in the healthy control group (127.13 ± 42.57 ng/mL), and significantly higher in the periodontitis group (277.73 ± 113.98 ng/mL) than in the gingivitis group (p < 0.001); thus, the periodontitis group had significantly higher salivary cortisol levels than the healthy control and gingivitis groups.

Salivary DHEA levels were significantly higher in the gingivitis (226.13 ± 56.60 pg/mL) and periodontitis (198.61 ± 35.03 pg/mL) groups than in the healthy control group (122.92 ± 24.35 pg/mL; p < 0.001). Furthermore, the cortisol/DHEA ratio was significantly higher in the periodontitis (1.41 ± 0.50) than in gingivitis group (1.06 ± 0.40; p < 0.001) and in the healthy control group (1.03 ± 0.20; p < 0.001). The salivary chromogranin A level was significantly higher in the gingivitis group (204.71 ± 34.20 ng/mL) than in the healthy control group (127.17 ± 46.68 ng/mL), and significantly higher in the periodontitis group (245.90 ± 69.31 ng/mL) than in the gingivitis group (p < 0.001).

Therefore, the stress-related neuroendocrine factors of cortisol, DHEA, cortisol/DHEA ratio, and chromogranin A were higher among patients with gingivitis and periodontitis than in healthy controls. Regarding the cortisol and chromogranin A levels, differences were observed even between the two periodontal disease groups (i.e., gingivitis and periodontitis groups; **Table 2**).

**Table 2:** Comparison of cortisol, dehydroepiandrosterone, chromogranin A, and cortisol/dehydroepiandrosterone ratio values by group.

	Healthy control (n=32)	Gingivitis (n=49)	Periodontitis (n=36)	p-value	*post-hoc* analysis
	mean ± SD	mean ± SD	mean ± SD		
**Cortisol (ng/mL)**	127.13 ± 42.57	231.52 ± 75.49	277.73 ± 113.98	<0.001***	Healthy control<Gingivitis, Healthy control<Periodontitis, Gingivitis<Periodontitis
**DHEA (pg/mL)**	122.92 ± 24.35	226.13 ± 56.60	198.61 ± 35.03	<0.001***	Healthy control<Gingivitis, Healthy control<Periodontitis
**Cortisol/DHEA ratio**	1.03 ± 0.20	1.06 ± 0.40	1.41 ± 0.50	<0.001***	Healthy control<Periodontitis, Gingivitis<Periodontitis
**Chromogranin A (ng/mL)**	127.17 ± 46.68	204.71 ± 34.20	245.90 ± 69.31	<0.001***	Healthy control<Gingivitis, Healthy control<Periodontitis, Gingivitis<Periodontitis

Analysis of variance and Tukey’s paired comparison test were used to compare the mean values among the three groups. Statistical significance was set at p < 0.05.

***: p < 0.001. The significant results are indicated in bold. DHEA, dehydroepiandrosterone.

### 3.3 Factors increasing cortisol, cortisol/DHEA ratio, and chromogranin A

Through multivariate logistic regression analysis, we investigated the significant predictors of increased cortisol level, cortisol/DHEA ratio, and chromogranin A level (**Table 3**). Among the 117 participants, the factors leading to above-average cortisol levels were periodontitis (OR = 256.829; p < 0.001), women (OR = 6.365; p = 0.004), and psychological stress (OR = 6.036; p = 0.007). In particular, periodontitis was found to be a very powerful biomarker for predicting above-average cortisol levels, whereas cortisol levels can be interpreted as the strongest factor explaining the existence of periodontitis.

**Table 3:** Multivariate logistic regression analysis of cortisol, cortisol/dehydroepiandrosterone ratio, and chromogranin A values.

Total sample (N=117)	Cortisol^+^				Cortisol/DHEA ratio^+^				Chromogranin A^+^			
	OR	95% Lower CI	95% Upper CI	p-value	OR	95% Lower CI	95% Upper CI	p-value	OR	95% Lower CI	95% Upper CI	p-value
Female [ref.=male]	**6.365**	1.831	22.130	0.004**	**2.890**	1.134	7.364	0.026*	1.188	0.477	2.958	0.711
Age [ref.:<average value]	0.351	0.107	1.156	0.085	0.607	0.229	1.610	0.316	1.056	0.415	2.688	0.910
Smoking habit [ref.=none]	0.802	0.107	6.039	0.831	3.044	0.459	20.166	0.249	3.130	0.467	20.962	0.240
Periodontitis [ref.=healthy control]	**256.829**	25.802	2556.484	<0.001***	**11.436**	2.925	44.722	<0.001***	**16.235**	4.103	64.237	<0.001***
Periodontitis [ref.=gingivitis]	0.551	0.158	1.926	0.351	1.916	0.699	5.250	0.206	**3.080**	1.096	8.654	0.033*
Xerostomia [ref.=SFR<2.0 mL/min]	**0.126**	0.027	0.585	0.008**	**0.346**	0.125	0.960	0.042*	0.455	0.161	1.288	0.138
Salivary pH [ref.:<average value]	0.241	0.033	1.772	0.162	2.703	0.713	10.248	0.144	0.619	0.162	2.363	0.482
Salivary buffer capacity [ref.:<10]	1.202	0.102	14.177	0.884	0.373	0.065	2.148	0.270	1.767	0.335	9.320	0.502
Psychological stress [ref.=none]	**6.036**	1.646	22.131	0.007**	**3.977**	1.609	9.828	0.003**	0.433	0.177	1.062	0.068

a The value of the parameter is above the average compared to when it is below the average. Results were obtained through multivariate logistic regression analysis. Statistical significance was set at p < 0.05. *: p < 0.05; **: p < 0.01; and ***: p < 0.001. The significant results are indicated in bold. CI, confidence interval; and DHEA, dehydroepiandrosterone.

The factors significantly predicting above-average cortisol/DHEA ratios were periodontitis (OR = 11.436; p < 0.001), psychological stress (OR = 3.977; p = 0.003), and women (OR = 2.890; p = 0.026). Factors predicting above-average chromogranin A values were periodontitis in healthy controls (OR = 16.235; p < 0.001) and periodontitis and gingivitis (OR = 3.080; p = 0.033). Accordingly, the above-average values of the three stress-related neuroendocrine factors were significantly affected by periodontitis, and only chromogranin A was significantly affected by both gingivitis and periodontitis. HPA axis activity was evidently different between the sexes, and female sex was identified as a main factor that significantly increased the cortisol levels and cortisol/DHEA ratios.

### 3.4 Correlations between the salivary stress-related hormonal factors

**Figure 2:**
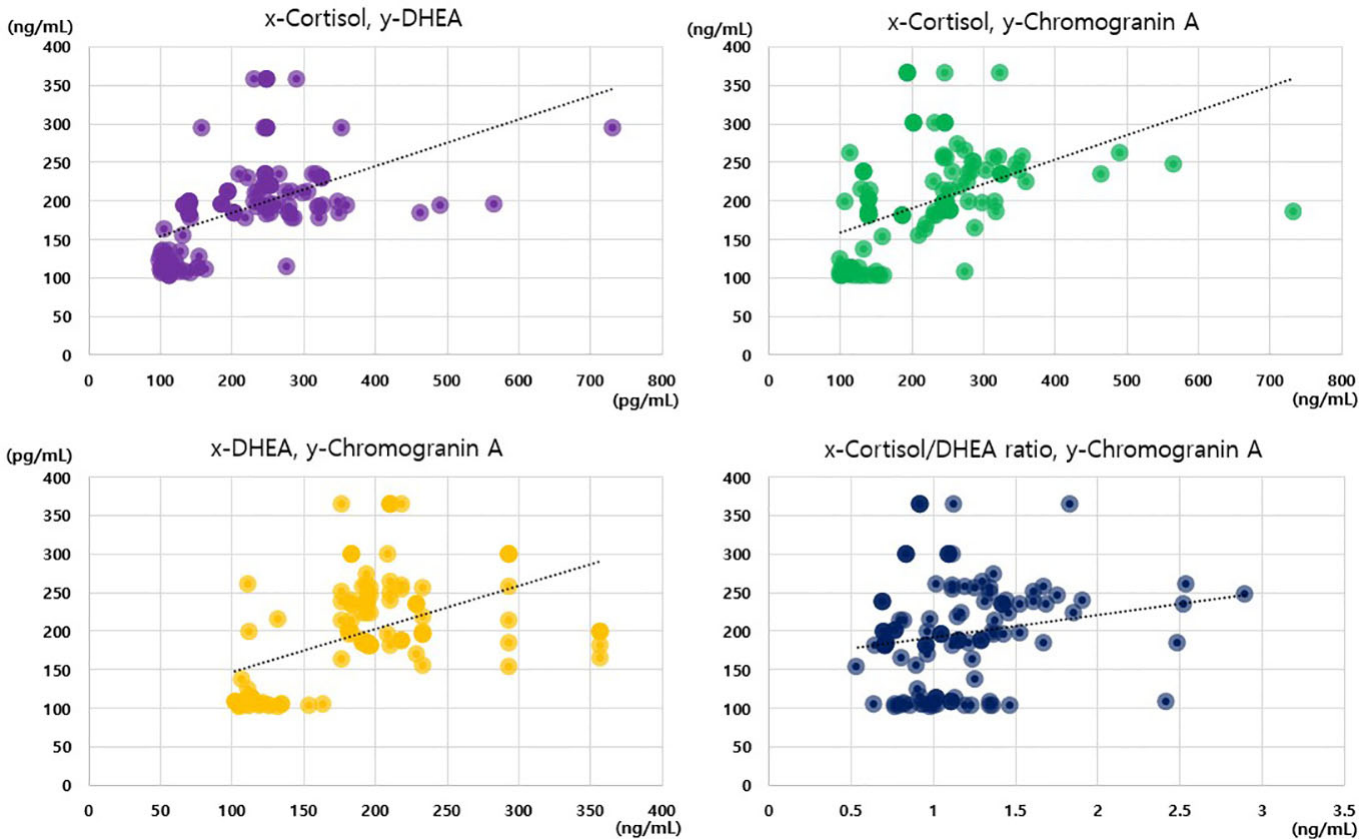
Correlations among cortisol, dehydroepiandrosterone, cortisol/dehydroepiandrosterone ratio, and chromogranin A values.

Notably, we only observed positive correlations between the cortisol, DHEA, and chromogranin A levels in the analysis of the data of the whole sample, and no such correlations were observed in the subgroup analyses. Among the 117 participants, we observed positive correlations between cortisol levels and cortisol/DHEA ratios (r = 0.668), cortisol and chromogranin A levels (r = 0.603), cortisol and DHEA levels (r = 0.559), and DHEA and chromogranin A levels (r = 0.505; all p < 0.001; **Figure 2**). The cortisol/DHEA ratio and chromogranin A levels were also positively correlated (r = 0.248, p = 0.007). The positive correlation was the strongest between cortisol levels and cortisol/DHEA ratios in the periodontitis group (r = 0.883; p < 0.001). We observed negative correlations between DHEA levels and cortisol/DHEA ratios in the healthy control and gingivitis groups but not in the periodontitis group (**Table 4**).

**Table 4:** Correlations among the hormonal parameters of salivary stress by group.

	Total			Healthy controls			Gingivitis			Periodontitis		
	DHEA	Cortisol/DHEA ratio	CgA	DHEA	Cortisol/DHEA ratio	CgA	DHEA	Cortisol/DHEA ratio	CgA	DHEA	Cortisol/DHEA ratio	CgA
**Cortisol**	**0.599**	**0.668**	**0.603**	0.129	**0.784**	0.182	0.207	**0.686**	0.249	0.167	**0.883**	-0.041
**DHEA**		-0.105	**0.505**		**-0.368**	-0.035		**-0.411**	-0.071		-0.202	0.046
**Cortisol/DHEA ratio**			**0.248**			0.252			0.135			-0.203

Results were obtained using Spearman’s correlation analysis. The significant results are indicated in bold. CgA, chromogranin A; and DHEA, dehydroepiandrosterone.

Red: positive correlation, blue: negative correlation, The darker the color, the greater the absolute value of the correlation coefficient.

### 3.5 Psychological stress and hormonal and salivary parameters

The factors that were correlated with psychological stress differed by group. In the healthy control group, psychological stress was significantly negatively correlated with decreases in salivary pH (r = -0.412, p = 0.019) and salivary buffer capacity (r = -0.387, p = 0.029). In the gingivitis group, psychological stress was significantly positively correlated with cortisol levels (r = 0.381, p = 0.007) and cortisol/DHEA ratios (r = 0.479, p < 0.001). In the periodontitis group, psychological stress was significantly correlated with an increase in cortisol/DHEA ratios (r = 0.412, p = 0.013) and a decrease in the salivary buffer capacity (r =-0.334, p = 0.047). These results show that the relationship between psychological stress and cortisol/DHEA ratio was not observed in the healthy control group but was observed in the two other groups, and that the relationship between psychological stress and cortisol levels was only observed in the gingivitis group. The relationship between the decrease in salivary buffer capacity and psychological stress was observed in the periodontitis and healthy control groups, but not in the gingivitis group. Notably, psychological stress was not correlated with either UFR or SFR in any of the three groups (**Table 5**).

**Table 5:** Correlation between psychological stress and hormonal and salivary parameters.

Correlation coefficient r with psychological stress		Cortisol	DHEA	Cortisol/DHEA ratio	Chromogranin A	UFR	SFR	Salivary pH	Salivary buffer capacity
**Healthy control**	**Psychological stress**	-0.303	0.021	-0.300	-0.010	0.063	-0.054	**-0.412**	**-0.387**
**Gingivitis**		**0.381**	-0.087	**0.479**	-0.218	0.037	0.077	-0.044	-0.236
**Periodontitis**		0.321	-0.027	**0.412**	-0.216	-0.271	-0.293	-0.188	**-0.334**

Results were obtained using Spearman’s correlation analysis. The significant results are indicated in bold. DHEA, dehydroepiandrosterone; SFR, stimulated salivary flow rate; and UFR, unstimulated salivary flow rate.

Red: positive correlation, blue: negative correlation, The darker the color, the greater the absolute value of the correlation coefficient.

## 4 Discussion

**Figure 3:**
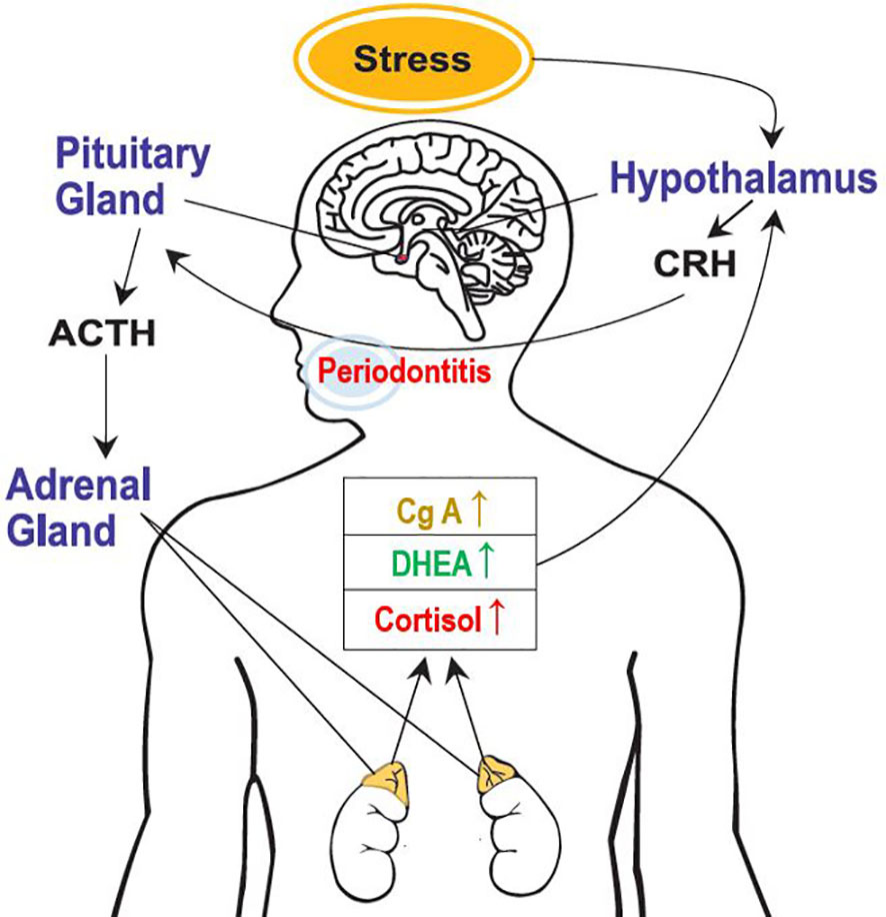
Activation of the hypothalamic-pituitary-adrenal axis because of psychological stress. CRH, corticotropin-releasing hormone; ACTH, adrenocorticotrophic hormone; DHEA, dehydroepiandrosterone; and CgA, chromogranin A.

This study clearly revealed the activation of the HPA axis due to psychological stress, a stress-related neuro-endocrine system, in patients with periodontal diseases and healthy participants (**Figure 3**). Moreover, salivary cortisol and chromogranin A levels increased with the severity of periodontal disease. The cortisol/DHEA ratio and DHEA levels were higher in the periodontitis group than those in the healthy control group. Notably, multivariate logistic regression analysis showed that periodontitis and psychological stress were significant predictors of above-average cortisol levels and cortisol/DHEA ratios. In patients with periodontal diseases, sex-differences may exist in the response of the HPA axis under stress. Compared with the male sex, the female sex showed significantly increased cortisol levels and cortisol/DHEA ratios. This study shows that the salivary characteristics and neuroendocrine parameters correlated with the presence of psychological stress differed by group. In particular, the relationship between psychological stress and cortisol/DHEA ratio was observed in the gingivitis and periodontitis groups, but not in the healthy control group. A significant positive correlation between psychological stress and salivary cortisol levels was observed only in the gingivitis group. Additionally, salivary cortisol and chromogranin A levels were more significant biomarker predictors for periodontitis and the severity of periodontal diseases. Furthermore, the DHEA, cortisol/DHEA ratio, cortisol, and chromogranin A values were more significant predictors of periodontitis than of healthy controls. Salivary cortisol levels could also predict the presence of psychological stress in the gingivitis group and predict cortisol/DHEA ratios in the gingivitis and periodontitis groups.

According to several statistical analyses performed in this study, salivary cortisol levels were the most consistent and strong biomarker predictors of periodontitis. In the literature, blood cortisol level has been considered as a relevant predictor of psychological stress. Blood cortisol levels were reflected in salivary cortisol levels within 2–3 min; thus, recent studies have been conducted on the value of salivary cortisol as a biomarker of psychological stress (40). Cortisol is a powerful anti-inflammatory hormone that mobilizes glucose for energy storage and regulates inflammation (41), and prolonged or exaggerated stress responses can lead to cortisol dysfunction and result in widespread inflammation and pain. Notably, upon comparing patients with chronic periodontitis with and without stress, researchers found increased salivary cortisol levels for those with stress (42). Additionally, a previous meta-analysis depicted that patients with aggressive periodontitis had higher salivary cortisol levels than healthy subjects and patients with chronic periodontitis (43). However, studies on the severity of periodontitis and related cortisol levels are very limited, and to the best of our knowledge, our study is the pioneering research to demonstrate that cortisol levels to be the highest in patients with periodontitis, followed by those with gingivitis and healthy controls. Previous studies have investigated salivary cortisol in patients with periodontitis, but there was no comparison with healthy controls (16, 42). Moreover, our study shows that the presence of psychological stress is associated with increased cortisol levels in patients with gingivitis. Cortisol is a well-known stress-related hormone that stably reflects the HPA activity, and has long been used as a biological marker of stress in human studies (44, 45). Combined with our results, cortisol can be used as a stress biomarker in patients with gingivitis and periodontitis. Additional long-term follow-up studies are required to further demonstrate the anti-inflammatory response of cortisol in patients with periodontal diseases.

In this study, DHEA levels and cortisol/DHEA ratios were higher in the gingivitis and periodontitis groups than among healthy controls. In previous human studies, the DHEA level, along with cortisol levels, was suggested as a major stress-related biomarker that increases with the HPA axis activity in response to stress (29, 46). In particular, studies have demonstrated that in response to stress, the adrenal gland co-releases DHEA and cortisol through a feedback loop initiated in the hypothalamus (47). Increased cortisol and DHEA levels are associated with increased PD and CAL in patients with periodontitis (48). Despite such evidence, in our study, although DHEA levels increased significantly in the following order: healthy controls, gingivitis, and periodontitis, the DHEA level was not a major predictor of psychological stress. Additionally, smoking habits did not affect cortisol levels or cortisol/DHEA ratios. Meanwhile, prior studies have reported that increased salivary hormone levels, including cortisol and DHEA levels, were greater in the patients who were smokers than those in the patients who were non-smokers (48), and that smoking clearly initiates and aggravates periodontitis (49). However, the relationship between smoking and psychological stress within the context of periodontal diseases has not been thoroughly elucidated, entailing the need for further research to clarify this relationship. Scholars have reported that serum cortisol levels and cortisol/DHEAS ratios were correlated with the severity of periodontitis in older adults (50). Other scholars have continued to use cortisol/DHEA ratios to examine the HPA activity and psychopathology (51, 52). This study suggests that an increased cortisol/DHEA ratio can be a predictor for both the presence of periodontitis and psychological stress in patients with gingivitis and periodontitis.

Chromogranin A is a glucoprotein released, along with catecholamines, in the adrenal medulla and sympathetic nerve endings, and its release is driven by precise coordination between the HPA axis and the sympathetic nervous system (53). Thus, this glucoprotein is recognized as a novel stress biomarker, particularly for examining psychological stress. In our study, chromogranin A levels significantly increased in the following order: healthy controls followed by patients with gingivitis and periodontitis; however, they were not a significant predictor of psychological stress in patients with periodontitis. Studies have shown that salivary chromogranin A is produced by the submandibular gland and secreted into the saliva following stimulation with noradrenaline and acetylcholine (54), and that chromogranin A levels were significantly higher in both saliva and plasma samples of patients with chronic periodontitis than in those of healthy individuals (55). Other researchers found significantly higher chromogranin A levels in the saliva samples of patients with aggressive periodontitis than in those of patients with chronic periodontitis and healthy controls (56). Therefore, numerous evidences suggest that chromogranin A is a potential novel biomarker of periodontitis. Meanwhile, our study was the first, to the best of our knowledge, to simultaneously measure cortisol and chromogranin A levels in patients with periodontitis. In a study examining occupational fatigue and hormonal changes, chromogranin A levels, but not cortisol levels, were correlated with occupational fatigue (57), and occupational stress was associated with higher levels of plasma chromogranin A (30). However, our results showed no significant relationship between psychological stress and chromogranin A in any group. Thus, our results suggest that chromogranin A could be a biomarker of gingivitis and periodontitis, although not biomarker of psychological stress in such patients.

Notably, periodontal disease is complex and is caused by a combination of several local and systemic pathologic, genetic, and socio-environmental factors. Primarily, periodontal disease occurs as a result of local infection with specific groups of pathogenic bacteria (58). However, a possible correlation between periodontal disease and systemic pathology, which has been considerably discussed in the scientific literature, should also be considered (33). In addition to oral infections and poor oral hygiene, risk factors, such as smoking, male sex, aging, medications, cardiovascular diseases, diabetes, rheumatoid arthritis, genetics, and psychological stress, have been associated with periodontal disease (59). In this study, female sex showed increased cortisol levels and cortisol/DHEA ratios. Periodontitis is more prevalent in men (~57%) than in women (~39%) (60). However, confounding factors affecting the development and progression of periodontal disease may differ between men and women (61). Further research is required to determine how psychological factors, such as stress and depression, intersect in periodontitis. Notably, periodontitis may trigger a systemic inflammatory response, or both of these may be bi-directionally correlated, and deserves more attention. Although our study was conducted in adults, periodontal diseases pose more risks for growing children and adolescents owing to the poor public health situation (62, 63). Several *in-vivo* and *in-vitro* studies of periodontitis have shown that the nervous and immune systems respond to systemic inflammatory responses, increasing interleukin-1, C-reactive protein, tumor necrosis factor, and matrix metalloproteinases (64, 65). Furthermore, psychological stress was associated differently according to negative psychosocial experiences, socioeconomic and behavioral factors, and sex (38). Thus, in patients with gingivitis and periodontitis, if the effects of emotional stress on the human body are evaluated based on the HPA axis perspective, errors can occur in the results or their interpretations.

This study had several limitations. First, we assessed the existence of psychological stress through a dichotomous evaluation, and not a quantitative one. The nature of the case-control study was added to this as participants could overreport on their moodiness rather than stress; conversely, if the survey was conducted in an uncomfortable environment, some potential biases might occur with the possibility of underreporting their stress. Further investigations are required to evaluate the existence of psychological stress in patients with periodontal disease as well as its duration, chronicity, precipitating factors, exacerbating factors, and clinical characteristics. With the binary question on psychological stress, there is inevitably a limit to sufficiently grasping and analyzing the patient’s stress. Additional analyses are required to clarify the relationship between the quantitative factors of psychological stress and neuroendocrine parameters. Additionally, although periodontal diseases include various conditions, we had divided them into the following two categories: gingivitis and periodontitis. Future studies must systematically examine both the physical and mental axes in patients with periodontitis.

In summary, this was the first study to simultaneously assess the cortisol level, DHEA, cortisol/DHEA ratio, and chromogranin A level in patients with periodontal diseases, comprising gingivitis and periodontitis, and compare the values of these markers with those of healthy controls. Furthermore, we studied the correlations among cortisol, DHEA, and chromogranin A levels and attempted to identify potent biomarker predictors of psychological stress in patients with gingivitis and periodontitis in comparison with those in healthy controls. However, the diagnosis of periodontitis is still a challenge (66). Our data on salivary biomarkers, namely salivary cortisol and chromogranin A levels and cortisol/DHEA ratio, can assist researchers and clinicians in overcoming the difficulties in the diagnosis of periodontitis, thereby increasing diagnostic accuracy.

## Data availability statement

The datasets presented in this study can be found in online repositories. The names of the repository/repositories and accession number(s) can be found in the article/**Supplementary Material**.

## Ethics statement

All participants were given information about the study and provided informed consent, and all protocols were approved by the Committee on Ethics of the Kyung Hee Clinical Research Institute, Kyung Hee University Medical Center (IRB no. KH-DT20030). The patients/participants provided their written informed consent to participate in this study.

## Author contributions

All authors including Y-HL, S-IS, and J-YH participated in writing and original draft preparation. Conceptualization, Y-HL; methodology, Y-HL; software, Y-HL; validation and formal analysis, Y-HL, S-IS, and J-YH; investigation, Y-HL, S-IS, and J-YH; resources, Y-HL, and J-YH; data curation, Y-HL; writing, review, and editing, Y-HL; visualization, Y-HL, CS, and J-YH; supervision, Y-HL; project administration, Y-HL; and funding acquisition, Y-HL. All authors contributed to the article and approved the submitted version.
